# Analysis of the motivational processes involved in university physical activity

**DOI:** 10.3389/fpsyg.2022.1080162

**Published:** 2023-01-09

**Authors:** Miguel Ángel Durán-Vinagre, Sergio J. Ibáñez, Sebastián Feu, Susana Sánchez-Herrera

**Affiliations:** ^1^Department of Psychology and Anthropology, Faculty of Education and Psychology, University of Extremadura, Badajoz, Spain; ^2^Department of Didactics of Musical, Plastic, and Corporal Expression, Faculty of Sport Science, University of Extremadura, Cáceres, Spain; ^3^Department of Didactics of Musical, Plastic, and Corporal Expression, Faculty of Education and Psychology, University of Extremadura, Badajoz, Spain

**Keywords:** motivation, self-determination, university students, physical activity, health

## Abstract

**Introduction:**

Physical activity plays an important role in all stages of development, especially in adolescence, as it is a period in which different lifestyles are shaped. Therefore, regular practice of physical activity contributes to an improvement in quality of life. This study analyzed university students’ motivational processes and intention to be physically active when engaging in physical activity (PA) according to gender and fields of study.

**Methods:**

A total of 1.524 subjects participated in the study, 61.9% (*n* = 944) of whom were female and 38.1% (*n* = 580) were male, with an average age of 19.61 years. The fields of study consisted of Arts and Humanities (*n* = 118), Science (*n* = 132), Health Sciences (*n* = 351), Engineering and Architecture (*n* = 196) and Social and Legal Sciences (*n* = 727). The instruments used were the Behavioral Regulation in Exercise Questionnaire (BREQ-3) and the Intention to be Physically Active in the University Context (MIFAU) measurement scale.

**Results:**

The results showed statistically significant differences in favour of men (*p* <0.01) in terms of intrinsic, integrated, identified, and introjected regulation of PA. Similarly, men were more likely to be physically active in the future than women (*p* <0.001). In terms of the study variables relating to the fields of study, statistically significant differences were obtained in the case of both more self-determined behavior (intrinsic, integrated and identified regulation) and amotivation (*p* <0.001).

**Conclusion:**

It was concluded that there is a need for the promotion of intervention strategies to encourage young adults to take up sporting activities as a means of preventing noncommunicable diseases, thereby avoiding the negative consequences of a sedentary lifestyle, physical inactivity, or abandonment of such activities.

## 1. Introduction

Physical activity (PA) has been applied to different areas of life, used through studies from motivation. Motivation is influenced by multiple and very diverse factors and is one of the most frequently studied aspects in the field of Sport Psychology, where it has been associated with various of the variables and agents involved (De Francisco et al., 2020).

The regular practice of PA by young people is considered beneficial to health as it can reduce the risk of chronic and non-communicable diseases, such as heart disease, cardiovascular accidents, diabetes, among others. It also highlights that this PA practice reduces symptoms of depression and anxiety (Chávez et al., 2018; Flores and Aceutino, 2021). According to the World Health Organization (WHO), by 2022 chronic diseases will account for 71% of the total number of deaths per year, with the percentage increasing in recent years in countries with fewer economic resources. Evidence suggests that chronic diseases are one of the greatest challenges facing health systems worldwide and they are strongly associated with several modifiable risk factors, notably physical inactivity, sedentary lifestyles and poor lifestyle habits (Anderson and Durstine, 2019; Ferrara et al., 2022).

In this sense, a large percentage of young people and adolescents do not reach the international recommendations of 60 min per day of moderate to vigorous intensity PA (Troiano et al., 2008). PA is essential as a natural protective factor for health as it is associated with numerous age-related health benefits, such as maintaining energy balance and preventing overweight and obesity, improving mental health and psychological well-being, increasing muscle strength and function, helping to promote positive mental health, among others (Kumar et al., 2015; Baran et al., 2020; Bull et al., 2020; Rodríguez et al., 2020). In the academic context, some studies point to the important role of educators to transfer healthy lifestyle habits from a very early age (De Craemer et al., 2013; Pulimeno et al., 2020). These educators are also responsible for the development of active and healthy behavior patterns that favor and facilitate the design of interventions aimed at increasing PA levels in students (Murillo et al., 2013). PA improvement and motivation in the university population is not only essential due to the low number of individuals who meet the recommendations, but also due to the fact that these students, especially those linked to health or education careers, can exert a major influence through their professional work, transmitting their lifestyle and promoting healthy habits (Mendoza-Núñez et al., 2013; Rippe, 2018).

This situation has made the study of the lifestyle of university students linked to these educational branches a topic of growing interest in the scientific literature (Tirodimos et al., 2009; Varela-Mato et al., 2012). A study by Molina de la Torre et al. (2012) indicated that university students linked to educational degrees in the field of Social and Legal Sciences and Health Sciences presented much lower levels of PA than those linked to the field of sports. In this line, a study by Cancela and Ayán (2011), in Spanish university students of Health Sciences and Education, showed a high prevalence of physical inactivity. Other studies suggest that men who pursue health-related careers tend to be more active than those linked to education or other professions, while this pattern does not occur in the case of women (Varela-Mato et al., 2012). The work of Farinola (2011) found that university students of Physical Education perform more PA than students of other university degrees.

In recent years, different theories have facilitated the understanding of motivational processes in the context of school and sport context, with Achievement Goal Theory (AGT) and Self-Determination Theory (SDT) standing out. The first of these, AGT, aims for the subject to be able to demonstrate his or her capabilities, where achievements may be guided by different conceptions that are affected by the social context (Ames, 1992; Moreno et al., 2007; Rottensteiner et al., 2015). SDT (Deci and Ryan, 2000) has been used to address motivation from the point of view of physical activity (Wang et al., 2009; Gómez-Mazorra et al., 2021). However, despite the emphasis on empirically studying the benefits linked to the promotion of healthy lifestyle habits, it is becoming increasingly difficult to achieve levels of physical activity appropriate to each age that not only provide positive and meaningful sporting experiences but also mitigate the problems of modern life, including sedentary lifestyles, physical inactivity and bad lifestyle habits associated with alcohol consumption, smoking and unhealthy eating (Deci and Ryan, 2000; Vázquez et al., 2019; Moral-García et al., 2020).

Some authors point out that a good alternative to carry out PA is to propose its promotion from the academic environment through SDT (Deci and Ryan, 1985; Zhang and Solmon, 2013). In this sense, SDT considers motivation as a fundamental element for the development of behavior, therefore, relating motivation and SDT will favor that people are motivated to grow and change due to basic psychological needs thanks to self-determination, i.e., their behaviors can be modified based on their regulation (Ryan and Deci, 2000a). In this sense, motivation should be oriented to serve the satisfaction of basic psychological needs, such as autonomy (feeling the cause of one’s actions), competence (perceiving oneself as capable of achieving specific goals) and relatedness (perceiving oneself as not emotionally isolated from others), which are key nutrients for the development of quality motivation and personal growth (Deci and Ryan, 2000; Ryan and Deci, 2017). In short, and as we well know, motivation plays an essential role in the practice and promotion of physical activity in all people, since human beings behave according to a series of motivational behaviors when it comes to achieving goals and objectives in different contexts (Rodríguez-Cañamero et al., 2010; Almagro et al., 2015).

According to its conceptualization of motivation, a psychological theory is only motivational if it takes into account the energy, direction, persistence and purpose of behavior, while at the same time considering the intentions behind certain resulting actions (Deci and Ryan, 1985). These authors considered SDT in relation to PA (Deci and Ryan, 1985; Deci and Ryan, 2000), proposing a self-determination continuum with the different types of motivation and their regulation styles. Depending on the satisfaction an individual obtains from doing physical activity, one type of motivation or another will come into play. These authors proposed a classification into intrinsic motivation, extrinsic motivation, and amotivation. The first mode involves engaging in activity for the pleasure deriving from its execution. Extrinsic motivation concerns behavior influenced by rewards and external agents. Finally, located at the other end of the self-determination continuum is amotivation, which refers to behavior not regulated by the subject, i.e., lack of intention to act (Deci and Ryan, 1985; Deci and Ryan, 2000).

More in-depth studies of motivation (Deci and Ryan, 1985; Chantal et al., 1996; Deci and Ryan, 2000) have identified four types of behavioral regulation associated with extrinsic motivation: integrated, identified, introjected and external regulation. Integrated regulation refers to conduct that is engaged in freely in and is present when the activity itself is immersed in the activity itself is immersed in the person’s lifestyle, in which characteristics that are which highlights characteristics related to values, goals, personal needs and identity (Deci and Ryan, 2000; Ryan and Deci, 2000a; Moreno and Martínez, 2006). Identified regulation differs from the rest in that it refers to highly-valued behavior that the individual identifies as important, i.e., the individual will perform the activity freely even if they do not enjoy it. It is a type of motivation in which the person perceives that the activity is carrying out is favorable, finding benefit from the fact of performing it, despite the fact that the person is not totally self-determined to perform it (Catarralá, 2004). This regulation of behaviors is autonomous, but the decision to participate in the activity is given by a series of external benefits and not by the pleasure and satisfaction inherent in the activity itself (Ntounamis, 2001). Introjected regulation is linked to expectations of self-approval, avoidance of anxiety or guilt and achievement of ego enhancement, such as pride. When participating in this type of activity, the main motive is social recognition, internal pressures or feelings of guilt (Ryan and Deci, 2000b). Finally, these authors understand external regulation as behavior in response to an external demand or the existence of rewards or compensation. Therefore, this regulation leads a subject to develop any activity for the reason of obtaining some kind of reward in return, without any kind of internalization, or for the consequent penalty of not developing it (Deci and Ryan, 2000).

Similarly, studies have shown there is a relationship between the practice of PA and more self-determined motivation (Moreno et al., 2007; DeFreese and Smith, 2013). The scientific literature supports the idea that more self-determined motivation is decisive to achieve greater beneficial effects from PA, such as control of body weight, increased physical fitness, positive improvement in mood, among others (Moreno-Murcia et al., 2011; Bermejo et al., 2018; Leyton et al., 2020). Studies by Durán-Vinagre et al. (2021) and Lauderdale et al. (2015) showed that there are statistically significant differences between men and women in terms of intrinsic motivation, integrated regulation and identified regulation. Furthermore, different studies suggest that the level of habitual physical-sports activity is higher in men than in women (López Sánchez et al., 2016; Li et al., 2017; Durán-Vinagre et al., 2019), with these levels decreasing progressively for both sexes as the years go by Sun et al. (2013), López Sánchez et al. (2016), and Li et al. (2017).

Different studies have concluded that PA plays a fundamental and irreplaceable role during adolescence because it is a stage characterized by the consolidation of patterns that will be maintained throughout an individual’s life (Litt et al., 2011; Varela et al., 2011; Kalman et al., 2015), and also because it contributes to the creation of healthy lifestyle habits as it is related to the transition to adulthood during which lifestyles are shaped (González et al., 2012). This may in turn be related to the concept of intention to be physically active in the future: according to Leyton et al. (2018), it is important to know the individual’s future intentions since beneficial behavior will lead to more effective healthy lifestyles accompanied by good practices, such as improving emotional well-being through PA, healthy eating, safety, and unintentional injuries, among others (Bonofiglio, 2022). Therefore, PA requires motivation to perform any bodily movement produced by skeletal muscles, with consequent energy consumption, as it determines the initiation, maintenance, and cessation of the conduct (Moreno et al., 2008).

Thus, although there are studies in different contexts in the scientific literature that use the psychological constructs outlined above with the university population (Práxedes et al., 2016; Leyton et al., 2018; Escamilla et al., 2020), to date there is no known research regarding PA in the university context, the motivational variables, and the intention to be physically active among first-year students, especially taking into account their different fields of study. This gap makes it difficult to argue this issue with the variables analyzed in the present study. Therefore, the main objective of this article is to analyze the motivational processes and the intention to be physically active of university students when engaging in PA according to gender and different fields of study.

## 2. Materials and methods

The method consisted of a non-experimental empirical study with a comparative research strategy using a cross-sectional (Ato et al., 2013) cohort design. The study used motivational variables and aspects associated with intention to be physically active in the future, taking into account gender and different fields of study.

### 2.1. Participants

A non-probability sample was selected of 1.524 first-year students from different degree courses of the University of Extremadura (Spain), of which 38.1% (*n =* 580) were male and 61.9% (*n =* 944) were female, with an average age of around 20 years (*M_age_* = 19.61 ± 3.65). The distribution of students by field of study was carried out according to the International Standard Classification of Education (ISCED) developed by the UNESCO Institute for Statistics (UNESCO, 2012) and was distributed as follows: Arts and Humanities 7.7% (*n =* 118), Science 8.7% (*n = *132), Health Sciences 23% (*n =* 351), Engineering and Architecture 12.9% (*n =* 196) and Social and Legal Sciences 47.7% (*n =* 727).

### 2.2. Instruments

Two questionnaires were used as measurement instruments for this study. The first of these was the Behavioral Regulation in Exercise Questionnaire (BREQ-3) in its Spanish version (Wilson et al., 2007; González-Cutre et al., 2010). This questionnaire consists of 23 items grouped into six factors, of which four belong to intrinsic motivation (e.g., “Because I find exercise an enjoyable activity”), four for integrated regulation (e.g., “Because I consider exercise to be part of me”), three for identified regulation (e.g., “Because I value the benefits of exercise”), four for introjected regulation (e.g., “Because I get nervous if I do not exercise regularly”), four for external regulation (e.g., “Because others tell me I should do it”), and four for amotivation (e.g., “I do not see why I have to do it”). The response scale was a Likert-type scale ranging from 0 (Not true at all) to 4 (Completely true). The factors and their reliability were: Intrinsic motivation (α = 0.90), Integrated regulation (α = 0.91), Identified regulation (α = 0.78), Introjected regulation (α = 0.70), External regulation (α = 0.80) and Amotivation (α = 0.78). The values of Cronbach’s alpha were mostly adequate (α > 0.70; Nunnally and Bernstein, 1994).

The second instrument used was the Intention to be Physically Active in the University Context (MIFAU) measurement scale, based on the Spanish version by Expósito et al. (2012). The questionnaire begins with the sentence “Regarding your intention to engage in physical-sporting activity.” and consists of five items, e.g., “I am interested in developing my physical condition.” The responses consisted of a Likert-type scale ranging from 1 to 5, with 1 = Strongly Disagree, 2 = Somewhat Disagree, 3 = Neutral, 4 = Somewhat Agree and 5 = Strongly Agree. The internal consistency value was α = 0.79 and it is therefore considered to have adequate reliability (α > 0.70; Nunnally and Bernstein, 1994).

### 2.3. Procedure

The study was conducted in accordance with the standards required by the American Psychological Association (2010). It was also approved by the Bioethics and Biosafety Committee of the University of Extremadura during its meeting held on 16 June, 2022. Permission was sought from both the lecturers responsible for the subjects taught in the different university degrees and from all the participants who completed the questionnaire. They were also informed that their participation was voluntary and anonymous in accordance with Spanish Law 15/1999 of 13 December on Protection of Personal Data. Before distributing the questionnaires, the objective of the survey was explained in detail, and it was indicated that the survey would take no more than 15 min to complete. The survey was conducted in person, with the principal investigator being present to collect the questionnaires. None of the participants reported any difficulties completing the instrument. The period for the implementation of the instrument was carried out during the academic year 2021/2022, adjusting to the availability and class times of the collaborating teachers and students.

### 2.4. Statistical analysis

Initially, the psychometric properties of the BREQ-3 and MIFAU scales were analyzed by means of a confirmatory factor analysis and internal consistency values expressed by Cronbach’s alpha.

Several indices (Hu and Benthler, 1999) were considered to evaluate the adequacy of the model fit of the questionnaires used (BREQ-3 and MIFAU): the global goodness-of-fit index (GFI), the adjusted goodness-of-fit index (AGFI), the normalized fit index (NFI), the relative fit index (RFI), the root mean square residual (RMR) and the standardized root mean square residual (SRMR). The values of the GFI, AGRFI, NFI and RFI range from 0 to 1, where 0 indicates no fit and 1 indicates optimal fit. Values of 0.95 or above are considered excellent and values above 0.90 suggest an acceptable fit of the model to the data. The optimal values of the fit for RMR and SRMR should be ≤0.1 (Hu and Benthler, 1995; Kline, 2010; Byrne, 2013). A model was then considered in which the regulators of motivation were related to the intention to be physically active, intrinsic motivation integrated and positively identified, while introjected, external and amotivation were negatively identified. The maximum likelihood (ML) method was implemented. The invariance of the model between gender groups was tested using multi-sample invariance analysis. Differences not greater than 0.01 in CFI (ΔCFI) and not greater than 0.015 in RMSEA (ΔRMSEA) were considered an indication of insignificant differences between groups (Cheung and Rensvold, 2002).

A descriptive analysis was also carried out to determine the nature of the data. Subsequently, the Mann–Whitney U test was used to obtain the gender differences between the variables studied and the Kruskal–Wallis *H* test was used for the fields of study. The significance level was set at *p<*0.05 (Newell et al., 2014). Finally, the Effect Size was calculated using Cohen’s *d*, classified as low effect (0–0.2), small effect (0.2–0.6), medium effect (0.6–1.2), large effect (1.2–2.0) and very large effect (>2.0; Hopkins et al., 2009). The software used was the SPSS 25 (Statistical Package for the Social Sciences, IBM Corp. Published in 2012. IBM SPSS Statistics for Windows, Version 25, IBM Corp, Armonk, NY, United States).

## 3. Results

Because the assumptions of normality were not met, the Unweighted Least Squares method was used for the confirmatory analysis of the Likert scales. The results indicated that the model had a good fit for the BREQ questionnaire (GFI = 0.99, AGFI = 0.98, NFI = 0.98 and RFI = 0.98) and that the values of the RMR (0.06) and SRMR (0.05) were adequate. In the case of the MIFAU questionnaire the indicators were also adequate (GFI = 0.99, AGFI = 0.99, NFI = 0.99, RFI = 0.98, RMR = 0.04 and SRMR = 0.03; Hu and Benthler, 1995; Kline, 2010; Byrne, 2013).

Table 1 shows the results of the descriptive analysis of the variables associated with the BREQ-3 and the MIFAU. Overall, the highest factors were intrinsic motivation and integrated regulation, with mean scores of 2.65 ± 1.10 and 2.18 ± 1.19, respectively. Meanwhile, amotivation and external regulation were the variables with the lowest scores, with means of 0.59 ± 0.79 and 0.45 ± 0.72, respectively. Regarding intention to be physically active in the future (MIFAU), the average value obtained was 3.76 ± 0.85, with the maximum score of the questionnaire being five points. Significant positive correlations were found between the intention to be physically active and the most self-determined, intrinsic, integrated, and identified types of regulation *p*<0.01.

**Table 1:** Descriptive results of the variables associated with the BREQ-3 and the MIFAU.

	Mean	SD	Variance	Skewness (Desv. Err = 0.06)	Kurtosis (Desv. Err = 0.12)	K-S	I	II	III	IV	V	VI
Intrinsic	2.65	1.10	1.23	−0.67	−0.40	0.12**						
Integrated	2.18	1.19	1.42	−0.10	−1.07	0.08**	0.76**					
Identified	2.11	0.72	0.52	−0.69	0.36	0.17**	0.49**	0.60**				
Introjected	1.12	0.89	0.80	0.61	−0.34	0.11**	0.23**	0.37**	0.46**			
External	0.45	0.72	0.52	1.89	3.39	0.29**	−0.27**	−0.16**	0.22**	0.30**		
Amotivation	0.59	0.79	0.64	1.65	2.76	0.23**	−0.44**	−0.40**	−0.30**	−0.08**	0.36**	
MIFAU	3.76	0.85	0.72	−0.69	0.04	0.10**	0.69**	0.70**	0.51**	0.28**	−0.21**	−0.44**

**p<0.01.

**Figure 1:**
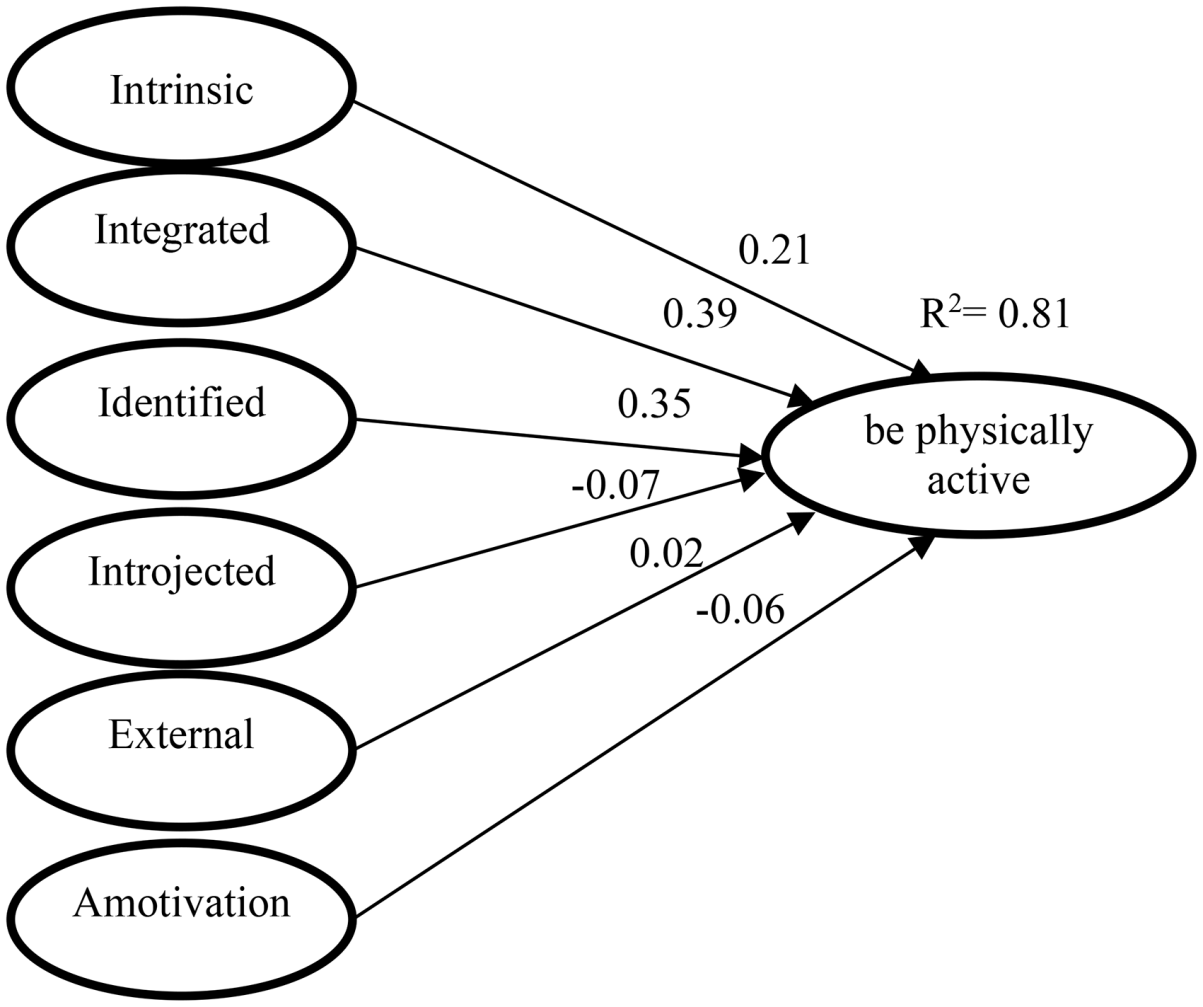
Model supporting regulators of motivation to be physically active.

Figure 1 shows the structural model, where the coefficient of determination is very high (*R*^2^ = 0.81), being greater than 0.3 (Chin, 1998). All factor loadings were greater than 0.50. The correlations between constructs are significant (*p*<0.001) in the case of intrinsic (β = 0.21), integrated (β = 0.39) and identified (β = 0.35) regulation, being the variables that predict being physically active. The *X*^2^/*df* ratio yields a result of 4.720 below 5, indicating an adequate fit of the data. The remaining measures also take values within the limits that allow us to affirm a good fit of the data (GFI = 0.93; AGFI = 0.91; CFI = 0.94; RMSEA = 0.05; NFI = 0.93; RMSEA = 0.49).

A multi-group confirmatory factor analysis (CFA) was performed to test the measurement invariance of the model as a function of sex. Initially, the baseline configuration invariance model (M1) was tested, where the *X*^2^/*df*, CFI, TLI and RMSA values ensured adequate fit (Table 2).

**Table 2:** Factor invariance across the gender variable.

Model	*X* ^2^	*df*	*X*^2^/*df*	*p*	CFI	TLI	RMSEA	AIC
M1 Configuration invariance (baseline)	1847.28	632	2.92	0.001***	0.94	0.93	0.036 (0.34–0.37)	2319.28
M2. Metric invariance	1925.12	653	2.94	0.001***	0.94	0.93	0.036 (0.34–0.38)	2355.12
M3 Scalar invariance	2156.13	681	3.16	0.001***	0.93	0.92	0.038 (0.34–0.38)	2530.13
M4. Strict invariance	2104.60	722	2.91	0.001***	0.92	0.92	0.037 (0.34–0.37)	2570.35

***p<0.001.

To obtain the metric invariance model, the regression weights or factor weights (M2) were added to the model. The values obtained are within the acceptable limits for goodness of fit (*X*^2^/*df* = 2.94; CFI = 0.94; TLI = 0.93; RMSEA = 0.03). Based on the nested model’s evaluation criterion, the CFI difference is less than 0.01 (0.003), and the RMSEA is less than 0.015 (0.000). To analyze the strong invariance (M3), the intercepts between groups were incorporated. The values obtained are within the acceptable limits for goodness of fit (*X*^2^/*df* = 3.16; CFI = 0.93; TLI = 0.92; RMSEA = 0.03). Based on the evaluation criteria for nested models, the CFI difference is less than 0.01 (0.009), and the RMSEA is less than 0.015 (0.002). Finally, strict invariance (M3) was analyzed, where the values *X*^2^/*df* = 2.91; CFI = 0.92; TLI = 0.92; RMSEA = 0.03 are within the limits for the sex variable. The variance of the AIC is not very high. Therefore, the goodness of fit of the model invariance of the compared models for the sex variable is verified (Cheung and Rensvold, 2002).

Motivational regulation and intention to be physically active were analyzed according to gender (Table 3). The results showed statistically significant differences in favour of men (*p*<0.01) in terms of intrinsic, integrated, identified, and introjected regulation of PA. The variable for the least self-determined behavior (amotivation) revealed more favorable results in the case of men, confirming the existence of significant gender differences (*p*<0.05). Men were also more likely to be physically active in the future than women (*p*<0.001). Finally, the analysis of the Effect Size found a medium effect for three of the variables analyzed: integrated (*d* = 0.481) and intrinsic (*d* = 0.394) regulation on the one hand, and the variable associated with the MIFAU scale on the other (*d* = 0.390).

**Table 3:** Inferential results of the regulation of motivation and intention to be physically active as a function of gender.

	Sex	Mean - SD	U	*p*	*d*
Intrinsic	Men	2.92 ± 0.41	210794.00	0.001***	0.394
	Woman	2.48 ± 0.03			
Integrated	Men	2.53 ± 0.04	197617.00	0.001***	0.481
	Woman	1.96 ± 0.03			
Identified	Men	2.20 ± 0.02	244654.50	0.001***	0.179
	Woman	2.05 ± 0.02			
Introjected	Men	1.21 ± 0.03	248678.50	0.003**	0.155
	Woman	1.06 ± 0.02			
External	Men	0.46 ± 0.02	265716.50	0.348	0.049
	Woman	0.44 ± 0.02			
Amotivation	Men	0.63 ± 0.03	257538.50	0.040*	0.100
	Woman	0.56 ± 0.02			
MIFAU	Men	3.95 ± 0.03	211460.50	0.001***	0.390
	Woman	3.64 ± 0.02			

*p<0.05; **p<0.01; ***p<0.001.

**Figure 2:**
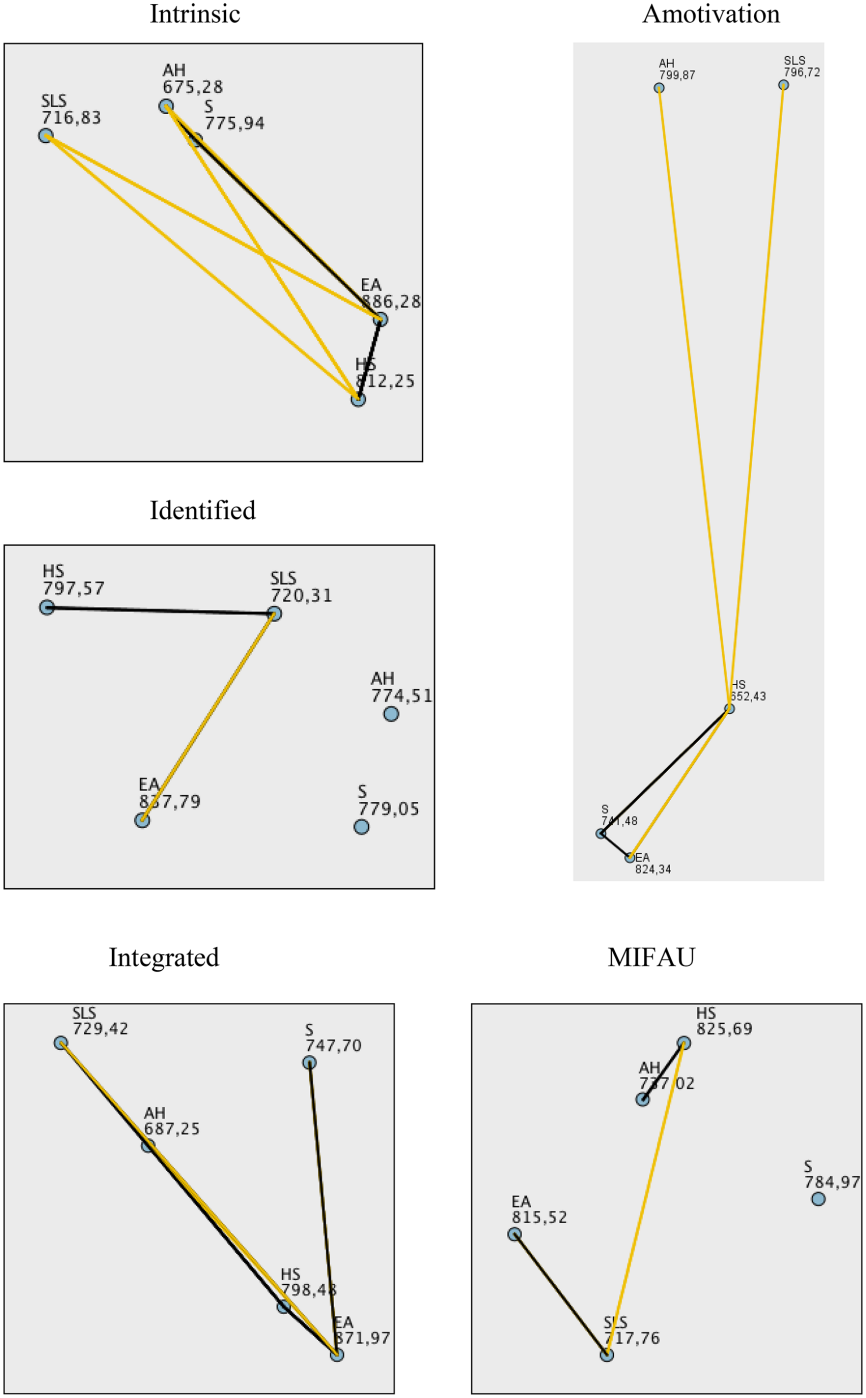
Multiple pairwise comparisons of exercise behavior regulation variables according to fields of study. AH, Arts and Humanities; S, Sciences; HS, Health Sciences; EA, Engineering and Architecture; SLS, Social and Legal Sciences.

Table 4 shows significant differences for several types of motivation according to the different fields of study, namely intrinsic motivation (*H* = 32.85; *p*<0.001), integrated regulation (*H* = 22.29; *p*<0.001), identified regulation (*H* = 15.40; *p*<0.01) and amotivation (*H* = 34.97; *p*<0.001). There were also statistically significant differences when analyzing intention to be physically active (*H* = 18.43; *p*<0.001). No significant differences were observed in the case of introjected regulation and external regulation (*p*>0.05; Figure 2).

**Table 4:** Inferential results of the regulation of motivation and intention to be physically active as a function of the field of study.

	*H*	*p*	*d*	Pairwise comparisons
Intrinsic	32.85	0.001***	0.278	EA > AH
Integrated	22.29	0.001***	0.221	EA > SLS
Identified	15.40	0.004**	0.174	EA > SLS
Introjected	3.41	0.491	0.039	-
External	8.78	0.067	0.112	-
Amotivation	34.97	0.001***	0.289	EA > HS
MIFAU	18.43	0.001***	0.196	HS > SLS

**p<0.01; ***p<0.001; AH, Arts and Humanities; S, Sciences; HS, Health Sciences; EA, Engineering and Architecture; SLS, Social and Legal Sciences.

## 4. Discussion

The aim of this study was to examine the differences in the types of motivation and the intention to be physically active among university students according to gender and different fields of study.

Our model findings show that the most self-determined behaviors (intrinsic, integrated and identified) predict intention to be physically active, the remaining behaviors (introjected, external and amotivation) do not. These data are in line with those obtained by other authors (Thøgersen-Ntoumani and Ntoumanis, 2006; Taylor et al., 2010; Esmaeilzadeh et al., 2022). We also found that there is no variance between genders, coinciding with other research showing that variables associated with PA are not consistent between genders (Azevedo et al., 2007).

The results indicate that the male students of the sample were characterized by greater intrinsic, integrated, identified, and introjected regulation than female students on the self-determination continuum. The results are similar to those of other studies, which found that individuals engaging in PA have high scores for these variables (Blázquez et al., 2015; Concha-Viera et al., 2017; Durán-Vinagre et al., 2022). This suggests a strengthening of the positive relationship between PA and more self-determined behavior (Concha-Viera et al., 2017).

On the other hand, the types of motivation that least characterized the study population were external motivation and amotivation. The same results were found in other studies, with low scores for these factors (Concha-Viera et al., 2017; Durán-Vinagre et al., 2021; Gutiérrez-García et al., 2021).

Differences were also found between men and women in terms of the different types of regulation. The results indicate statistically significant differences for intrinsic, integrated, identified, introjected regulation, and amotivation with the mean differences for men standing out with respect to women. The studies by Durán-Vinagre et al. (2022) and Luque-Casado et al. (2021) revealed similar results, with the men in the sample being significantly more motivated than the women. One of the possible explanations for this significant gender difference may be that it is more culturally acceptable for the male role to be characterized by more stimulating, dynamic, and active activities than the female role (Concha-Viera et al., 2017; Martínez and Sauleda, 2019).

In terms of the study objectives, it can be seen that the sample showed statistically significant differences in terms of intention to be physically active in the future, with men scoring more favorably for this variable. In contrast to these findings, (Durán-Vinagre et al., 2019; Sánchez-Herrera et al., 2022) obtained the same statistical differences in terms of gender.

If we focus on the university years, women studying at Spanish universities have more sedentary lifestyles with low levels of physical activity. The main causes are the consumption of unhealthy foods, high levels of stress and anxiety and little free time due to the academic commitments of current degree courses (Díaz et al., 2014). These causes are considered by other authors to be the most frequent barriers to making academic tasks compatible with physical and sporting activity (Martínez-Lemos et al., 2014; Samperio et al., 2016; Castañeda et al., 2018).

Finally, the different types of motivational regulation and intention to be physically active were related to the different fields of study (Arts and Humanities, Science, Engineering and Architecture, Health Sciences and Social and Legal Sciences). Significant differences were found for these variables in terms of intrinsic, integrated and identified regulation, amotivation, and intention to be physically active. In contrast to these results, (Sánchez-Herrera et al., 2022) found that Health Sciences students showed a more favorable tendency towards intrinsic and integrated regulation and lower scores for external regulation and amotivation. Likewise, these results support those of Gutiérrez-García et al. (2021) given that the same differences were obtained when comparing the means. However, there is a lack of studies in the scientific literature that relate the variables studied with different fields of study, making it more difficult to compare the results obtained. There is research such as Práxedes et al. (2016), Chung et al. (2018), Sevil et al. (2018), Durán-Vinagre et al. (2019), and Durán-Vinagre et al. (2020) that consider the types of motivation or intentionality related to PA in university students from different specific university degrees or studies that do not specify the characteristics of the participants in the sample, without extracting and comparing results that are related to participants from other university degrees that are within the same field of knowledge.

This gap allows us to speculate on our findings, and we can deduce that students of Health Sciences are more likely to show a more intrinsic motivation, being able to value more the benefits and improvements that encompass motivation and PA from a healthy point of view. However, it is striking that students of Engineering and Architecture present a great difference with those of Arts and Humanities. These data are associated with the values obtained for amotivation, i.e., the lack of interest in doing PA, so that on this occasion it is the students of Engineering and Architecture who show a significant difference with respect to those of Social Sciences and Law. Finally, when considering the intentionality of being physically active, the students of Health Sciences show a greater interest in practicing sports than those of Social and Legal Sciences. These are data that should be contrasted with further research in the future in order to respond to similar empirical studies with another university population sample.

The results for the study population underline the importance of continuing with this line of research to generate information favoring the development of strategies that contribute to more self-determined regulation in the practice of PA. Resources, tools and other means are needed to promote values that are important for university students and thereby achieve greater enjoyment of regular physical activity in any of its forms. In this sense, it would be advisable to promote recreational activities that allow physical activity to be seen as an activity freely engaged in and not an institutionalized or compulsory activity.

## 5. Conclusion

The findings show that men are characterized by more self-regulated behavior than women. These results also offer an insight into the types of behavioral regulation of physical activity among university students in different fields of study. They are a valuable tool when it comes to establishing intervention strategies for the prevention of noncommunicable diseases that take into account the peculiarities of the university context and the possibility of promoting and facilitating physical-sports activity among university students according to their diversity.

Key activities need to be identified that represent an enjoyable and positive experience for these individuals. University students have many difficulties adhering to PA programs, but if we know their characteristic psychological variables, strategies can be proposed that take into account the improvement of physical condition and also the creation of healthier lifestyle habits that allow them to maintain more regular PA. Efforts should also be made to ensure this group adheres to sporting activities and does not abandon them. University sport services should promote an adequate environment and a range of physical-sporting activities to favor more self-determined motivation, allowing students to appreciate the benefits of being physically active and thereby mitigating the consequences deriving from physical inactivity.

## Data availability statement

The original contributions presented in the study are included in the article/supplementary material, further inquiries can be directed to the corresponding author.

## Ethics statement

The studies involving human participants were reviewed and approved by Research Bioethics Committee of the University of Extremadura and received a favorable ethical opinion (code: 95/2022). The participants provided their written informed consent to participate in this study.

## Author contributions

MÁD-V, SF, SJI, and SS-H conceived the aim of this study. MÁD-V participated in the data collection. MÁD-V, SF, and SJI participated in the methods and results. All authors have contributed to the writing-review and editing of the article and have also read and accepted the published version of the manuscript.

## Funding

This study has been partially funded by the Research Grants Group (GR21157 and GR21149) of the Regional Government of Extremadura (Regional Ministry of Economy, Science and Digital Agenda), with a contribution from the European Regional Development Fund: A way of doing Europe (ERDF). It has also been financed by the Plan Own Initiation to Research, Technological Development and Innovation of the University of Extremadura 2021.

## Conflict of interest

The authors declare that the research was conducted in the absence of any commercial or financial relationships that could be construed as a potential conflict of interest.

## Publisher’s note

All claims expressed in this article are solely those of the authors and do not necessarily represent those of their affiliated organizations, or those of the publisher, the editors and the reviewers. Any product that may be evaluated in this article, or claim that may be made by its manufacturer, is not guaranteed or endorsed by the publisher.
